# Arginyl-tRNA-protein transferase 1 contributes to governing optimal stability of the human immunodeficiency virus type 1 core

**DOI:** 10.1186/s12977-021-00574-0

**Published:** 2021-09-26

**Authors:** Naoki Kishimoto, Ryosuke Okano, Ayano Akita, Satoshi Miura, Ayaka Irie, Nobutoki Takamune, Shogo Misumi

**Affiliations:** 1 Department of Environmental and Molecular Health Sciences, Faculty of Medical and Pharmaceutical Sciences, Kumamoto University, Kumamoto, 862-0973 Japan; 2 Kumamoto Innovative Development Organization, Kumamoto University, Kumamoto, 860- 8555 Japan

**Keywords:** Human immunodeficiency virus type 1, Uncoating, HIV-1 core, Arginyl-tRNA-protein transferase 1

## Abstract

**Background:**

The genome of human immunodeficiency virus type 1 (HIV-1) is encapsulated in a core consisting of viral capsid proteins (CA). After viral entry, the HIV-1 core dissociates and releases the viral genome into the target cell, this process is called uncoating. Uncoating of HIV-1 core is one of the critical events in viral replication and several studies show that host proteins positively or negatively regulate this process by interacting directly with the HIV-1 CA.

**Results:**

Here, we show that arginyl-tRNA-protein transferase 1 (ATE1) plays an important role in the uncoating process by governing the optimal core stability. Yeast two-hybrid screening of a human cDNA library identified ATE1 as an HIV-1-CA-interacting protein and direct interaction of ATE1 with Pr55^*gag*^ and p160^*gag − pol*^ via HIV-1 CA was observed by cell-based pull-down assay. ATE1 knockdown in HIV-1 producer cells resulted in the production of less infectious viruses, which have normal amounts of the early products of the reverse transcription reaction but reduced amounts of the late products of the reverse transcription. Interestingly, ATE1 overexpression in HIV-1 producer cells also resulted in the production of poor infectious viruses. Cell-based fate-of-capsid assay, a commonly used method for evaluating uncoating by measuring core stability, showed that the amounts of pelletable cores in cells infected with the virus produced from ATE1-knockdown cells increased compared with those detected in the cells infected with the control virus. In contrast, the amounts of pelletable cores in cells infected with the virus produced from ATE1-overexpressing cells decreased compared with those detected in the cells infected with the control virus.

**Conclusions:**

These results indicate that ATE1 expression levels in HIV-1 producer cells contribute to the adequate formation of a stable HIV-1 core. These findings provide insights into a novel mechanism of HIV-1 uncoating and revealed ATE1 as a new host factor regulating HIV-1 replication.

**Graphic abstract:**

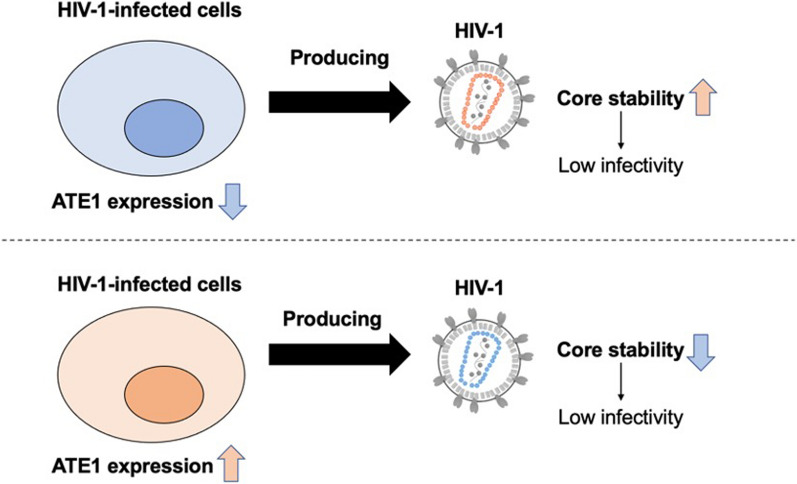

## Background

Human immunodeficiency virus type 1 (HIV-1) capsid proteins (CA) compose a conical core to encapsulate the viral genome. The monomer CA assemble as a hexamer or pentamer to form an optimal shape containing ~ 250 hexamers and 12 pentamers [1]. In complete HIV-1 replication, assembled CA should disassemble at viral target cells to release the viral genome. This step is called uncoating. Importantly, although optimal uncoating of the viral core is required for successful infection, the detailed machinery of uncoating is still controversial. Tang et al. [2] and Forshey et al. [3] demonstrated that mutations in various CA amino acids affect the HIV-1 core morphology and optimal stability, and decrease reverse transcription efficiency. Importantly, Forshey et al. [3] also demonstrated that either increase or decrease core stability reduces viral infectivity. We previously reported that peptidyl-prolyl isomerase Pin1 promotes uncoating by recognizing the phosphorylated Ser^16^-Pro^17^ motif outside the HIV-1 core [4]. Furthermore, several studies have identified CA interacting proteins that regulate uncoating, such as nucleoporin 153 (Nup153), Nup358, cleavage and polyadenylation specificity factor 6 (CPSF6), transportin-1, cyclophilin A (CypA), alpha isoform of tripartite motif protein 5, and myxovirus resistance 2 [5–14]. It has also been reported that the viral core uncoats in the nucleus, and the importance of proteins involved in core translocation, such as Nup153 and CPSF6, has been confirmed [15, 16]. These findings indicate that the stability of the HIV-1 core has an enormous impact on viral replication and host proteins can modulate the stability of the HIV-1 core. Therefore, sufficient characterization of CA-interacting proteins is required for resolving uncoating machinery.

We identified arginyl-tRNA-protein transferase 1 (ATE1) as a novel protein that interacts with CA region of viral precursor proteins. ATE1 mediates protein arginylation, one of the protein posttranslational modifications, and transfers Arg from tRNA^arg^ to the acceptor protein. Several studies demonstrated that ATE1 attaches Arg to Asp, Glu and Cys of *N*-terminally exposed amino groups [17–20], and the side chains of Asp and Glu of midchain residues [21, 22]. Importantly, ATE1 shows biological roles in processes involving the *N*-end rule pathway that governs the rate of protein degradation [17–19], protein stability and protein-protein interactions [23, 24], and endoplasmic reticulum stress responses [25, 26]. These findings indicate that ATE1 may contribute to cellular fate and the functional regulation of intracellular HIV-1 Pr55^*gag*^ and CA.

In this study, we newly found that ATE1 is a new protein that directly binds to the *N*-terminal domain (residues 1-151) of the CA. Decreased expression levels of ATE1 in HIV-1 producer cells resulted in the production of less infectious viruses, which have normal amounts of the early products of the reverse transcription reaction but reduced amounts of the late products of the reverse transcription. In addition, altered expression levels of ATE1 in HIV-1 producer cells resulted in the reduction of the optimal stabilized HIV-1 core formation regardless of the uptake of major viral structural proteins in the produced virus particles. This is the first report that ATE1 modulates HIV-1 replication.

## Results

### ATE1 was identified as a protein that interacts with CA region of viral precursor proteins

**Fig. 1:**
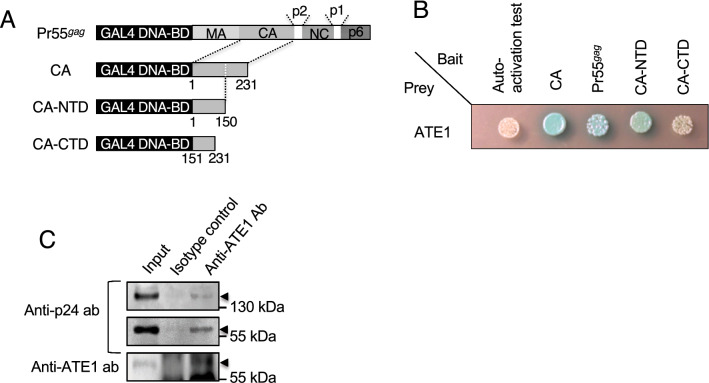
ATE1 interacts with HIV-1 Pr55^*gag*^ and CA-NTD. **A** Bait constructs obtained from pNL-CH are illustrated. **B** Y2H analysis of ATE1 with CA, Pr55^*gag*^, CA-NTD or CA-CTD. The Y2HGold strain was cotransformed with ATE1 as prey protein and viral proteins as bait proteins. Growth on QDO/X/A plates with blue colonies indicates a positive interaction. Shown are data from three experiments with a similar outcome. **C** Determination of ATE1-Pr55^*gag*^ and p160^*gag − pol*^ interaction. ATE1 was immunoprecipitated from the clarified lysate from CEM/LAV-1 cells with anti-ATE1 antibody and co-immunoprecipitated proteins were detected using anti-p24 antibody

We performed yeast two-hybrid (Y2H) screening to identify host factors that contribute to HIV-1 uncoating. In the screening, the cDNA sequences of positive clones revealed that the CA interacted with ATE1. To validate the interaction between the CA and ATE1, some viral genes containing the CA were expressed as a fusion to the GAL4 DNA-binding domain (Fig. 1A) and the ATE1 gene was expressed as a fusion to the GAL4 activation domain. The constructed bait and prey vectors were cotransformed into the yeast strain (Y2HGold) and the yeast was grown on QDO/X/A (quadruple dropout medium lacking adenine, histidine, tryptophan and leucine, and supplemented with X-α-Gal and aureobasidin A) plates. As a result, the growth of blue colonies on the QDO/X/A plates indicated the positive interaction between the CA and ATE1 (Fig. 1B). In addition, the assay showed that HIV-1 Pr55^*gag*^ and the CA *N*-terminal domain (CA-NTD) interacted with ATE1, suggesting that the *N*-terminal domain (residues 1-150) of the CA was responsible for interaction with ATE1. To confirm the interaction between HIV-1 precursor proteins, which containing CA region, and ATE1 in cells, we performed a co-immunoprecipitation assay using an anti-ATE1 antibody. As a result, we detected a positive interaction between ATE1 and Pr55^*gag*^ and p160^*gag − pol*^ (Fig. 1C). Since CA is cleaved from Pr55^*gag*^ by processing step, our results suggested that ATE1 interacts with Pr55^*gag*^ and p160^*gag − pol*^, especially through the *N*-terminal region of CA.

### ATE1 knockdown cell-produced virus shows low infectivity

**Fig. 2:**
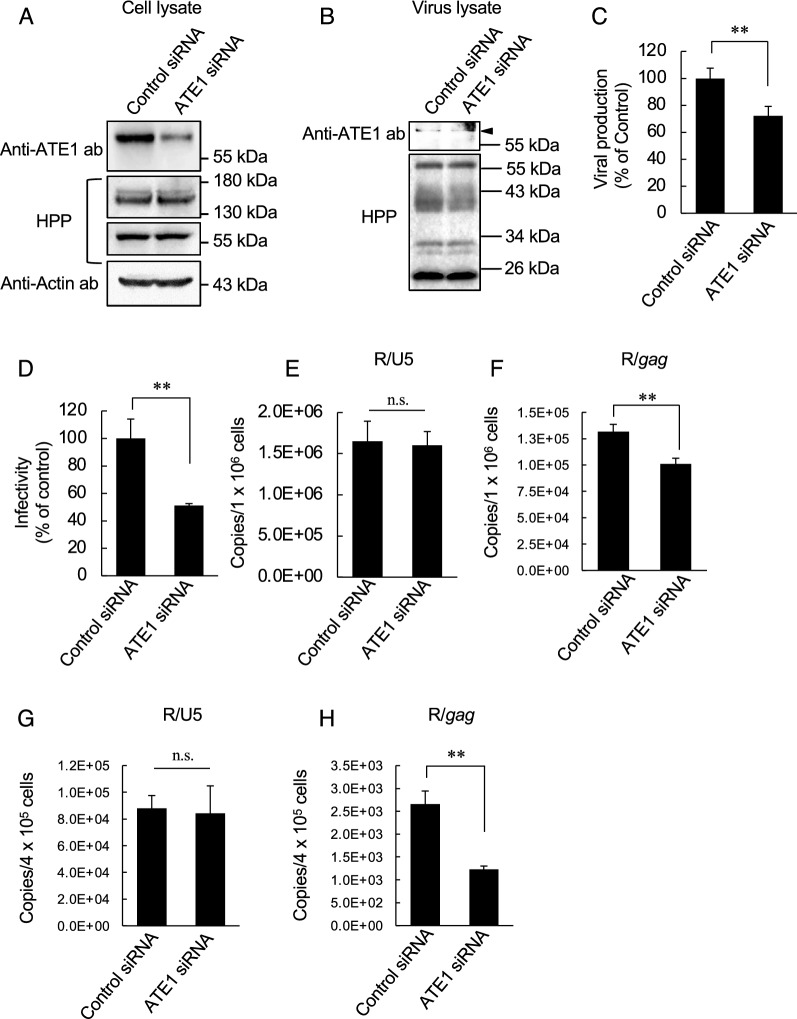
ATE1 knockdown in HIV-1 producer cells decreases the infectivity of progeny viruses. **A** Confirmation of ATE1 knockdown efficiency. CEM/LAV-1 cells were transfected with control or ATE1 specific siRNA. ATE1 expression levels were confirmed by western blotting at 48 h post-transfection. **B** Effect of ATE1 knockdown in viral producer cells on the packaging of viral proteins in progeny viruses. The purified viruses were subjected to western immunoblotting. Each viral protein was detected by HIV-1-positive plasma. The arrow indicates the positive band of ATE1. **C** Effect of ATE1 knockdown in viral producer cells on viral production. Viral production levels were monitored by p24 ELISA of culture supernatant of transfected cells. The value in the control experiment was set as 100 %. **D** Infectivity of viruses produced from control or ATE1 specific siRNA-treated cells. TZM-bl cells were incubated with an equal amount (1 ng of p24 antigen) of each virus. The infectivity was examined based on the luciferase activity in the lysates of each virus-infected TZM-bl cell sample. The value in the control experiment was set as 100%. **E** Amount of de novo synthesized R/U5 cDNA products of viruses produced from control or ATE1 specific siRNA-treated cells in TZM-bl cells. TZM-bl cells were incubated with an equal amount (10 ng of p24 antigen) of each virus. **F** Amount of de novo synthesized R/*gag* cDNA products of viruses produced from control or ATE1 specific siRNA-treated cells in TZM-bl cells. TZM-bl cells were incubated with an equal amount (10 ng of p24 antigen) of each virus. **G** Amount of de novo synthesized R/U5 cDNA products of viruses produced from control or ATE1 specific siRNA-treated cells in PBMCs. PBMCs were incubated with an equal amount (10 ng of p24 antigen) of each virus. **H** Amount of de novo synthesized R/*gag* cDNA products of viruses produced from control or ATE1 specific siRNA-treated cells in PBMCs. PBMCs were incubated with an equal amount (10 ng of p24 antigen) of each virus. The significance of difference (Student’s *t*-test) is indicated as follows: **p < 0.01; *n.s.* not significant. The mean values of at least three independent experiments are shown. The error bars denote the standard deviation

To investigate the contribution of ATE1 in HIV-1 replication, we transfected a chronically HIV-1-infected T-cell line (CEM/LAV-1 cells) with control siRNA or ATE1-specific siRNA and measured the infectivity of progeny viruses. As shown in Fig. 2A, ATE1 siRNA significantly decreased ATE1 expression levels in CEM/LAV-1 cells without altering the expression levels of HIV-1 precursor proteins (Pr55^*gag*^ and p160^*gag − pol*^), and as shown in Fig. 2B, ATE1 knockdown did not affect the packaging and processing levels of viral proteins in virions. Interestingly, western immunoblotting of purified viruses revealed that ATE1 was incorporated into viral particles and the amount of integrated ATE1 appeared to be dependent on its expression level in virus producer cells (Fig. 2B). Although the expression levels of viral proteins, such as Pr55^*gag*^, in viral producer cells did not affect by ATE1 knockdown, the viral production levels were decreased (Fig. 2C). In addition, progeny viruses from ATE1-knockdown cells showed lower infectivity than those from control siRNA-treated cells (Fig. 2D). To further characterize the effects of ATE1 knockdown in CEM/LAV-1 on progeny viruses, we evaluated the reverse transcription steps. We quantified the amount of reverse transcription product after infection of TZM-bl cells or peripheral blood mononuclear cells (PBMCs) with each virus. We found no difference in the amount of early reverse transcription product (Fig. 2E, G), but the amount of late reverse transcription product was decreased in the virus produced from ATE1-knockdown cells (Fig. 2F, H). These results indicate that down-regulation of ATE1 expression in HIV-1 producing cells leads to the production of less infectious viruses with impairment at the step of late reverse transcription.

### ATE1 overexpression cell-produced virus also shows low infectivity

**Fig. 3:**
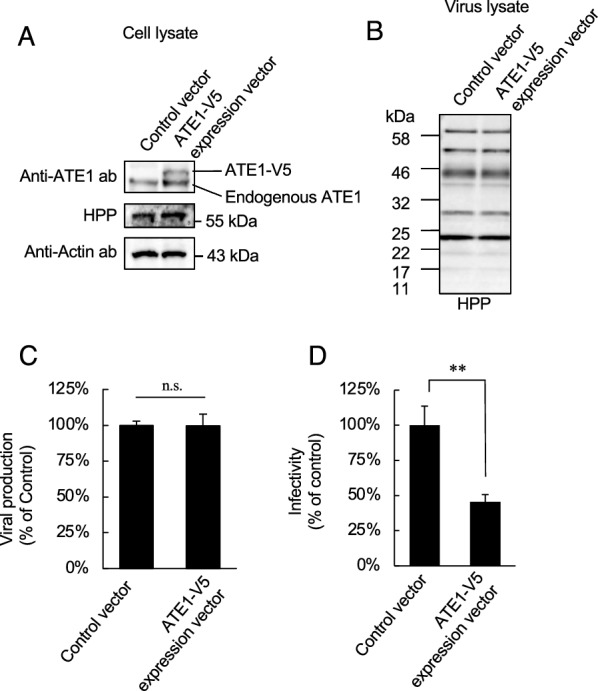
ATE1 overexpression in HIV-1 producer cells decreases the infectivity of progeny viruses. **A** Confirmation of ATE1 overexpression efficiency in viral producer cells. HEK293 cells were cotransfected with pNL-CH and control or ATE1-V5 expression vector. **B** Effect of ATE1 overexpression in viral producer cells on the packaging of viral proteins in progeny viruses. The purified viruses were subjected to western immunoblotting and each viral protein was detected by HIV-1-positive plasma. **C** Effect of ATE1 overexpression in viral producer cells on viral production. HEK293 cells were cotransfected with pNL-CH and the ATE1-V5 expression vector. Viral production levels were monitored by p24 ELISA of culture supernatant of transfected cells. The value in the control experiment was set as 100%. **D** Infectivity of viruses produced from control or ATE1 specific siRNA-treated cells. The infectivity was examined based on the luciferase activity in the lysates of each virus-infected TZM-bl cell sample. The value in the control experiment was set as 100%. The significance of difference (Student’s *t*-test) is indicated as follows: **p < 0.01; *n.s.* not significant. The mean values of at least three independent experiments are shown. The error bars denote the standard deviation

To further investigate the contribution of ATE1 in HIV-1 replication, we prepared and evaluated viruses produced from ATE1-overexpressing cells (Fig. 3A). As shown in Fig. 3B, ATE1 overexpression did not affect the packaging and processing levels of viral proteins in virions. In addition, ATE1 overexpression did not affect viral production (Fig. 3C). However, the viruses produced from ATE1 overexpression cells showed lower infectivity than those produced from control cells (Fig. 3D). These results indicate that the alteration of ATE1 expression levels in HIV-1 producer cells induces the production of less infectious viruses.

### ATE1 expression levels in HIV-1 producer cells modulate the stability of HIV-1 core

**Fig. 4:**
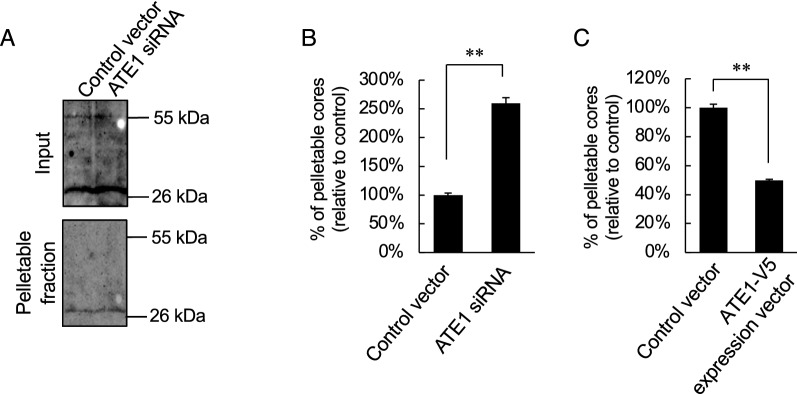
ATE1 expression levels in HIV-1 producer cells influence the core stability of progeny viruses. **A**–**C** Effect of ATE1 expression levels in viral producer cells on the fate of CA core of progeny viruses [(**A**, **B**) the viruses produced from ATE1-knockdown cells, (**C**) the viruses produced from ATE1 overexpression cells]. Fate-of-capsid assay was performed in accordance with the method of Sodroski [36]. The amount of CA cores presented in the cytosol was analyzed Western blotting (**A**) or ELISA (**B** and **C**) for the CA proteins. The value in the control experiment was set as 100%. The significance of difference (Student’s *t*-test) is indicated as follows: **p < 0.01; *n.s.* not significant. The mean values of at least three independent experiments are shown. The error bars denote the standard deviation

Since Forshey et al. demonstrated that altered CA core stability decreased viral infectivity, we speculated that the alteration of ATE1 expression levels in HIV-1 producer cells influences the core stability. To examine whether core stability was altered by ATE1 expression levels, we carried out a cell-based fate-of-capsid uncoating assay. The western immunoblotting showed that the intensity of the CA band tends to increase in the pelletable fraction of the viral capsids obtained from cells treated with ATE-1 siRNA. In contrast, at the input, the Pr55^*gag*^ signal was weak, and there was no difference in the amount of Pr55^*gag*^. Since CA-containing Pr55^*gag*^ species were not detected in the pelletable fraction, the amount of pelletable CA cores were measured by ELISA. As shown in Fig. 4B, the amounts of pelletable CA cores in the TZM-bl cells infected with the viruses produced from ATE1-knockdown cells were higher than those produced from the control virus-infected cells (Fig. 4B). In contrast, the amounts of pelletable CA cores in the cells infected with viruses produced from ATE1-overexpressing cells were lower than those produced from the control virus-infected cells (Fig. 4C). These results indicate that alteration of ATE1 expression levels in HIV-1 producer cells abrogates HIV-1 replication by preventing the formation of a stable CA core suitable for virus replication and optimal uncoating.

## Discussion

Optimal uncoating of the viral core is required for successful HIV-1 replication, but its mechanism and requirement of cofactors have remained poorly defined. To date, there have been reports of CA point mutations that reduce or increase the stability of the HIV-1 CA core. In this study, we identified ATE1 as a novel regulator of uncoating. Importantly, both low and high ATE1 expression levels in HIV-1 producer cells changed core stability in the target cell and impaired HIV-1 infectivity without altering the quality of major viral constituent proteins. These results support the idea that the formation of a virus core with optimal stability is a prerequisite for efficient HIV-1 infection and depends at least on the expression level of ATE1 in HIV-1 producer cells.

We clarified that ATE1 interacts with the CA domain of HIV-1 precursor proteins and governs viral uncoating. Several studies demonstrate that host protein-dependent post-translational modifications of CAs are involved in the stability of the core [4, 27–29]. Because ATE1 regulates various biological processes through arginylation and has been reported to be involved in protein stability and protein-protein interactions [23, 24], we speculated that ATE1 induces CA arginylation and performed mass spectrometry analysis to identify arginylated residues in the CA protein. The CA in viral particles were composed of at least four isoforms of the CA proteins with different isoelectric points [4]. The peptide fragments derived from these CA isoforms have been analyzed by mass spectrometry, but so far, the arginylation site in the CA has not been identified. On the other hand, we were able to detect ATE1 in viral particles and the expression of the putative active site mutant (ATE1 C71S) in ATE1-overexpressing cells rescued the viral infectivity to the same extent as control experiment levels (unpublished data). Therefore, we speculated that the enzymatic activity of ATE1 in viral producer cells is important for the arginylation of the CA region of Pr55^*gag*^ to form a stable CA core and that arginylated CA is required for HIV-1 infection.

CA needs to undergo post-translational modifications such as phosphorylation for viral uncoating [30, 31]. The CA Ser^16^ phosphorylation has a significant effect on core stability and uncoating [4]. We reported that Ser^16^ is phosphorylated in viral particles by extracellular signal-regulated kinase 2 [27], and Takeuchi et al. [28] reported that Ser^149^ is phosphorylated by maternal embryonic leucine zipper kinase required for optimal uncoating of the HIV-1 core. Furthermore, Cartier et al. [31] reported that CA Ser^109^ and Ser^178^ phosphorylation are also essential for viral uncoating process. However, the enzymes involved in the phosphorylation of Ser^109^ and Ser^178^ have not yet been identified. The CA core may have a positive charge due to arginylation, but the charge balance on the surface of the CA core may be controlled by the sequential phosphorylation of Ser residues. In the future, we would like to clarify the more detailed uncoating process by determining the arginylation site of CA by ATE1 and investigating whether ATE1 is involved in the functional regulation of unknown kinases involved in the phosphorylation of Ser^109^ and Ser^178^.

## Conclusions

Although optimal uncoating is required for successful HIV-1 replication, its detailed uncoating mechanism is still controversial. Here, we identified ATE1 as a new host factor involved in HIV-1 uncoating. The alteration of ATE1 expression levels in HIV-1 producer cells abrogated optimal uncoating and reduced the viral infectivity. We propose that the suitable ATE1 expression levels in HIV-1 producer cells are required for the optimal uncoating of the HIV-1 CA core to maintain HIV-1 infectivity.

## Methods

### Cells

TZM-bl cells were obtained from the NIH AIDS Research and Reference Reagent Program. CEM/LAV-1 or TZM-bl cells and HEK293 cells were maintained at 37 °C in RPMI-1640 or DMEM supplemented with 10% fetal bovine serum containing 100 IU/ml penicillin and 100 µg/ml streptomycin in 5% CO_2_. PBMCs were isolated using Ficoll-Paque (Pharmacia Biotech, Piscataway, NJ) density gradient centrifugation, as previously described [32]. PBMCs were cultured in medium containing concanavalin A and IL-2 for 72 h before being used for infection experiments.

### Plasmids

The infectious molecular clone pNL-CH [33], which was derived from the pNL_4−3_ clone of HIV-1, was a gift from R. Swanstrom. To perform Y2H screening, the Pr55^*gag*^ and CA coding region of the HIV-1_NL–CH_ were cloned into pGBKT7 (bait) (Clontech Laboratories, Inc.) as previously described [34].To prepare the ATE1-V5 expression vector, the coding region of ATE1, which was amplified by PCR using the primers ATE1-TCF (5′-AAAGCTTGCCATGGCTTTCTGGGCGGGG-3′) and ATE1-TCR (5′-TCTCGAGCAGTTTCTGAACAGCAGCATCCG-3′), was cloned into the pcDNA™ 3.1D/V5-His-TOPO^®^ vector (Thermo Fisher Scientific, Inc.).

### Y2H analysis

The Matchmaker™ Gold Yeast Two-hybrid System (Clontech Laboratories, Inc.) was used in accordance with the manufacturer’s recommendations for library screening. To carry out human cDNA library screening, the HeLa S3 (normalized) library encoded by the pGADT7 AD vector (Clontech Laboratories, Inc.) was selected. The library cDNA inserts were sequenced and proteins determined using the NCBI BLAST program. Cotransformation assay was performed as previously described [34]. Briefly, each viral protein (bait) and ATE1 derived from the Hela S3 cDNA library (prey) were cotransformed into Y2HGold and grown on QDO/X/A plates. The autoactivity of ATE1 (autoactivation test in Fig. 1A) was confirmed by transforming only the prey vector into Y2HGold in the absence of the bait vector. The autoactivity of each viral protein was previously tested [34].

### Transfection

CEM/LAV-1 cells were transfected with 100 nM validated commercially available Silencer™ ATE1 siRNA (Catalog #:43,90,824, Thermo Fisher Scientific Inc.) to suppress ATE1 expression using the Neon™ transfection system (Thermo Fisher Scientific Inc.) [32, 35]. To overexpress ATE1, cells were transfected with the control vector or ATE1-V5 expression vector using Lipofectamine^®^ LTX reagent and Plus™ reagent (Thermo Fisher Scientific Inc.). All experiments were performed in accordance with the manufacturer's instructions. The expression level of each protein was determined by western immunoblotting using an anti-ATE1 antibody (Santa Cruz Biotechnology, Inc.), an anti-V5 antibody (Thermo Fisher Scientific Inc.), an anti-actin antibody (Wako Pure Chemical industries, Ltd.), or HIV-1-positive plasma (a gift from Dr. Matsushita, Kumamoto University).

### Co-immunoprecipitation

CEM/LAV-1 cells were lysed in low-salt lysis buffer (1% Igepal CA630, 50 mM Tris HCl (pH 8.0). The soluble lysate was pre-cleaned with µMACS™ Protein G MicroMeads (Miltenyi Biotec) without any antibody, and then incubated with anti-ATE1 antibody or isotype control mouse IgG_2a_ antibody for 30 min at 4 ºC, and further incubated with 50 µL of µMACS™ Protein G MicroMeads (Miltenyi Biotec). Immunoprecipitated complex was separated by µ Columns (Miltenyi Biotec). Pr55^*gag*^ and p160^*gag − pol*^ were detected using anti-p24 antibody.

### Viruses

Viruses produced from CEM/LAV-1 cells were prepared from culture supernatants of CEM/LAV-1 cells, as previously described [32]. Viruses produced from pNL-CH were prepared from culture supernatants of pNL-CH-transfected HEK293 cells. To determine infectivity, the viral titer was normalized by the p24 level, which was calculated by p24 ELISA (ZeptoMetrix Corporation). To detect intravirion proteins, the supernatant from the culture medium was centrifuged at 43,000×*g* for 3 h at 4 °C following filtration with a 0.22-µm-pore size filter. The obtained pellet was washed with PBS(−) (0.02% KH_2_PO_4_, 0.29% Na_2_HPO_4_·12H_2_O, 0.8% NaCl, 0.02% KCl) and then centrifuged at 1,00,000×*g* for 1 h at 4 °C. The washing step was repeated three times. The resulting pellet was subjected to western immunoblotting.

### Evaluation of viral infection

Viral infectivity was determined by measuring the luciferase activity in TZM-bl cell lysates, and *de novo* synthesized viral cDNA products were measured by quantitative real-time PCR analysis as described previously [32]. To measure viral infectivity, TZM-bl cells were incubated with an equal amount (1 ng of p24 antigen) of each virus. To measure the levels of de novo synthesized viral cDNA products, TZM-bl cells or PBMCs were incubated with an equal amount (10 ng of p24 antigen) of each virus. The amounts of R/U5 and R*/gag* products were amplified using the following primers: R/U5 products, M667 (5′-GGCTAACTAGGGAACCCACTG-3′) and AA55 (5′-CTGCTAGAGATTTTCCACACTGAC-3′); R/*gag* products, M667 and M661 (5′-CCTGCGTCGAGAGAGCTCCTCTGG-3′).

### Fate-of-capsid assay

Fate-of-capsid assay was performed by previously described methods with some modifications [4, 29, 36]. TZM-bl cells were infected with each virus sample (25 ng of p24 antigen) at 37 °C for 2 h following 0.5 h of incubation at 4 °C. The cells were washed twice with PBS(-) and treated with 500 µL of pronase at 4 °C for 10 min. Purified cells were homogenized and centrifugated as previously described. After centrifugation, each fraction was collected and the amount of the p24 antigen in each fraction was measured by p24 ELISA (ZeptoMetrix Corporation). In adddtion, the pelletable fraction was detected by western immunoblotting.

## Data Availability

All data generated or analyzed during this study are included in this published article.

## Abbreviations

HIV-1: Human immunodeficiency virus type 1
CA: Capsid proteins
ATE1: Arginyl-tRNA-protein transferase 1
PBMCs: Peripheral blood mononuclear cells
Y2H: Yeast two-hybrid
CA-NTD: CA *N*-terminal domain
CA-CTD: CA *C*-terminal domain
Nup: Nucleoporin
CypA: Cyclophilin A
CPSF6: Cleavage and polyadenylation specificity factor 6
